# Current state of infection and prevalence of giardiasis in Malaysia: a review of 20 years of research

**DOI:** 10.7717/peerj.12483

**Published:** 2021-11-11

**Authors:** Norhamizah Roshidi, Nur Hassanah Mohd Hassan, Asma Abdul Hadi, Norsyahida Arifin

**Affiliations:** Institute for Research in Molecular Medicine, Universiti Sains Malaysia, Penang, Malaysia

**Keywords:** Giardiasis, prevalence, Malaysia, Diarrheal disease, Waterborne infection

## Abstract

**Background:**

Giardiasis is a neglected parasitic zoonotic disease caused by *Giardia duodenalis* that is often overlooked despite the damage inflicted upon humans and domestic/wild animals. Lack of surveillance studies, low sensitivity of diagnostic tools, and resistance to giardiasis treatment add to the challenge in managing giardiasis, leaving a gap that continues to render giardiasis a silent threat to public health worldwide. This situation is not much different in Malaysia, where giardiasis remains a public health problem, especially in the indigenous communities. Realizing the existence of gaps in the literature and information on giardiasis in Malaysia, this review aims to revisit and update the situation of giardiasis in Malaysia based on articles published in 20 years from 2000 to 2020, providing estimates on the incidence of giardiasis in humans, animals, and the environment, which may inform efforts to prevent and control the impact of giardiasis in the country.

**Methodology:**

We searched PubMed, Science Direct, and Scopus using MeSH terms and text keywords “*Giardia duodenalis* OR *Giardia intestinalis* OR *Giardia lamblia* OR intestinal protozoa AND Malaysia”. Information was collected from all giardiasis reports published between 2000 and 2020.

**Results:**

Giardiasis in Malaysia is more prevalent among the poorest segments of the population, namely the indigenous communities and people living in densely populated areas such as slums and prisons, due to low standard of personal hygiene, unsafe water resources, and improper sanitation. While the prevalence data is hugely dependent on microscopic fecal examination in epidemiological studies of giardiasis, current studies mostly focused on species identification and genotype distribution by multilocus genotyping. Thus far, the outbreak of giardiasis has not been reported in the country, but the disease was found to be significantly associated with stunting, wasting, and malnutrition among children of the indigenous communities. Surveillance studies also discovered the simultaneous presence of *Giardia* in the animal-environments, including wild animals, ruminants, and treated and untreated water. The data collected here will be a useful addition to the literature body on giardiasis in Malaysia, which can be exploited in efforts to prevent and control the impact of giardiasis in the country.

**Conclusions:**

The last 10 years have shown that the overall mean rate of giardiasis in Malaysia is quite encouraging at 13.7%. While this figure appears to be declining, there has been a slight increase in the prevalence of underweight, stunting, and wasting among rural children in 2019. The fact that giardiasis is linked to long-term childhood developmental problems, indicates that addressing and providing better disease control against giardiasis should be a priority in supporting the national agenda to achieve Malaysia Global Nutrition Targets by 2025.

## Introduction

Diarrheal disease is defined as one of the top ten causes of mortality and disability-adjusted life-years for persons in all age groups, hence placing diarrhea as an important threat in the global health radar. There were about 1.6 million cases of diarrheal deaths reported in 2016, of which about 90% of deaths occurred in South Asia and sub-Saharan Africa (Troeger et al., 2018). Significantly, one in nine child deaths are due to diarrhea, contributing to 2,195 deaths per day, bringing the total to 801,000 child deaths every year, more than the combined cases of malaria, measles, and AIDS (Centers for Disease Control and Prevention, 2015). Approximately 250 million of the reported diarrheal cases include people affected with giardiasis, a parasitic disease caused by *Giardia duodenalis* (syn. *G. lamblia* and *G. intestinalis*) (Adam, 2001). With diarrhea as the second leading cause of infectious diseases-related morbidity and mortality after pneumonia among children, it is important to control the progression of giardiasis so as to reduce the likelihood of an increase in diarrhea-related deaths (Farthing et al., 2013). Clean water and good sanitation are the two-pronged strategies to address giardiasis–which is hardly present and severely impact the children, health, and socioeconomics of those living in disadvantage community settings. Consequently, this has led to the inclusion of giardiasis in the WHO Neglected Diseases Initiative in 2004 (Savioli, Smith & Thompson, 2006). Not only that, giardiasis is also of significant clinical and economic importance in livestock and pet animals, demanding One Health’s integrated approach to comprehensively control giardiasis.

The last few decades have witnessed that *Giardia* has been elevated from its place as a commensal to that of an important pathogen. The fact is that *G. duodenalis* is considered a complex species with high diversity, where immunological mechanisms, disease manifestations and pathophysiology are still poorly understood and understudied, let alone the parasitic virulence characteristics and the determinants of the hosts’ responses that remained clearly undefined, leaving clear gaps that need deep focus and in-depth studies on giardiasis. Therefore, this review aims to revisit and update the situation of giardiasis in Malaysia, with emphasis on disease prevalence in humans, animals, and the environment, as well as the associated risk factor based on studies published in the country from the past 20 years. The data collected here will be a useful addition to the literature body on giardiasis in Malaysia, providing information to clinicians, health providers, researchers, and politicians in efforts to prevent and control the impact of giardiasis in the country.

### Search strategy

We searched PubMed, Science Direct and Scopus using MeSH terms keywords “*Giardia duodenalis* OR *Giardia intestinalis* OR *Giardia lamblia* OR intestinal protozoa AND Malaysia”. The search was restricted to articles published within the last 20 years (2000–2020), where all relevant abstracts, systematic reviews and articles reporting data on epidemiology, occurrence, clinical manifestations, diagnosis, interventions and genotyping in Malaysia were screened and heeded. Articles that do not match the keywords stated in the title or the abstract were removed from the study list, including studies with confusing information. The list of publications was then exported to Excel in CSV format and multiple entries were then de-duplicated.

### Global incidence of giardiasis

The worldwide prevalence of giardiasis has been reported to be as high as 20–40% in low-income countries, with the risk being greatest among young children under 5 years of age, and 2–7% in high-income countries, where almost 40% of cases occurred in travelers returning from highly endemic areas (Feng & Xiao, 2011). Poverty and poor hygienic conditions clearly promote the transmission of giardiasis in areas without adequate access to hygiene and sanitation infrastructure, whereas in developed areas with higher sanitary standards, the disease is perceived as a reemerging infection being commonly associated with traveling and presumably contaminated water supplies. Giardiasis is also the cause of various foodborne diseases and day care center outbreaks through transmissions via direct fecal–oral route, zoonotic, or person-to-person contact, with immunocompromised individuals, young children, and patients with cystic fibrosis being among the high-risk group (Lalle & Hanevik, 2018).

Out of 199 protozoan-waterborne outbreaks that reportedly occurred worldwide from 2004 to 2010, *Giardia* was the etiological agent in 35.2% (70) of the outbreaks (Baldursson & Karanis, 2011). Meanwhile from 2011 to 2016, at least 381 outbreaks attributed to waterborne transmission of parasitic protozoa were documented, and *Giardia spp.* were mentioned in 37% (142) of the cases (Efstratiou, Ongerth & Karanis, 2017). The serious impact of the epidemic on public health rendered giardiasis to be a national notifiable infection in many countries *i.e.*, the United States, the United Kingdom, Canada, and Australia for public health surveillance. From 1995 to 2016, a total of 435, 186 cases of giardiasis were reported to the National Notifiable Diseases Surveillance System (NNDSS), of which 5.1% of the cases were reported as outbreak-associated, with an incidence rate of 6.4 cases per 100,000 populations in 2016 (Coffey et al., 2021). Canada reported 19.6 cases of giardiasis per 100,000 people in 2015, whereas in 2017, South West of England had the highest rate of *Giardia* with 20.1 laboratory reports per 100,000 populations (The Government of Canada, 2019; gov.uk, 2018). While giardiasis is not part of the national health surveillance registers in Denmark, it is systematically reported in Norway and Sweden and is a voluntary-based registration system in Finland (Hörman et al., 2004). These Nordic countries reported a number of 0.35–16.06 cases per 100, 000 populations annually. Interestingly, the incidence rate was surprisingly high in New Zealand, which is reported to be at 44.1 per 100,000 populations and is considered as one of the highest among developed countries (Snel, Baker & Venugopal, 2009). The rates reported in other countries are 1.3%–38% (Iran), 6.5% (Saudi Arabia), 73.4% (Nepal), 3–50% (Mexico), 37.7% (Thailand), and 24.9% (Malaysia) (Feng & Xiao, 2011).

However, these estimations could be underestimated due to several limiting factors such as reliance on low sensitivity parasitological diagnosis resulting in the inability to detect asymptomatic and subclinical cases, lack of surveillance studies, and no authoritative reporting activity, all of which play significant roles in case detection, infection transmission, and mapping the epidemiology. Despite the high burden and significant impact on humans and animals, giardiasis is not considered a health priority due to lack of political will, funding, and interest from scientific community. It was not until 2004 that the interest in research and publication associated to giardiasis has raised due to the inclusion of giardiasis in the Neglected Diseases Initiative by the WHO (Savioli, Smith & Thompson, 2006) and because of the growing recognition of its role as a cause of disease outbreaks.

### Giardiasis in Malaysia

Our primary searching on giardiasis in Malaysia identified 68 articles from PubMed database, 148 from Scopus and 61 from Science Direct, giving a total of 277 articles published from 2000 to 2020. After careful screening based on the title and abstract along with the removal of duplicates, only 62 articles were related and included in the present review article.

In the context of the situation in Malaysia, much of the literature and research activities focused on epidemiological studies and the distribution of genetic diversity in specific populations of the indigenous communities (Orang Asli), children, immunocompromised patients, and migrant population. In the early 1970s, the prevalence data of giardiasis in Malaysia relied on fecal surveys of intestinal parasitic infections (IPIs) for which giardiasis is one of the targets. Research to date tends to focus on epidemiological studies targeting the same marginalized populations with the inclusion of molecular characterization and multilocus genotyping by PCR and RFLP-PCR to further identify the genotype and subgenotype of *G. duodenalis*. Such information is important to better understand the genetic diversity and dynamic transmission, to facilitate in outbreak surveillance, to trace for contamination source-tracking and to unveil the potential of zoonotic transmission.

### Prevalence of giardiasis in humans

Drawing on an extensive range of research sources, Sinniah et al. (2014) collated a comprehensive data on the prevalence of IPIs among communities living in different localities in Malaysia. From 1970 to 2013, the prevalence of IPIs among Orang Asli was reportedly high, accounting to 87.3%–44.3%, so as the rural communities (90%–32.3%), the slum dwellers (95%–20.6%), and fishing communities (93.8%–54.2%), while urban and flat dwellers reported a lower prevalence of 35.8%–45.2% and 19.3%–5.5%, respectively (Sinniah et al., 2014). While these rates were seen to fluctuate over 42 years, it was reported to have declined in the last 10 years with an overall mean of 13.7%, and this improvement is partly influenced by the success of intervention programs through mass drug administration under the national helminth control program undertaken by the Ministry of Health Malaysia, in addition to improved basic amenities, income, better healthcare and lifestyle (Sahimin et al., 2020).

A closer look into giardiasis infection showed that from 1970 to 1987, cases of giardiasis in Malaysia reportedly ranged from 2.6% to 25% (Kan, 1988; Lai, 1992), at the rate of 5.2%–19.1% from 1982 to 1992 (Lai, 1992), and 1.4%–11.1% from 1992 to 1994 (Shekhar, Prathapa & Gurpreet, 1996). Shekhar, Prathapa & Gurpreet (1996) also analyzed the prevalence data from 1970 to 1994 and reported a prevalence range of 2.6%–25%, with a higher percentage found in rural communities where both basic amenities and safe water supply are insufficient and lacking. While details on cases of giardiasis reported between the 1970s and 2000 can be found in Shekhar, Prathapa & Gurpreet (1996), Norhayati et al. (2003) and Sinniah et al. (2014), this review aims to highlight the trend of giardiasis cases in Malaysia from 2000 to 2020, emphasizing on the spread of the disease among the community, the animals, and the environment.

Within 20 years, a total of 32 publications were identified to report the prevalence of giardiasis among the Malaysian population, of which 84.3% reported cases occurred among the indigenous people particularly children, 12.5% were hospital-based studies and the rest were studies that focused on other population *i.e.*, migrants and urban communities (Table 1). Data on the prevalence of giardiasis for the year 2000 can be referenced in two retrospective studies of hospital records by Mohamed et al. (2008) and Nissapatorn et al. (2005); the former found 55 positive giardiasis cases from 9992 stool samples collected from 2000 to 2006 and the latter reported 6 cases out of 1350 samples from samples collected from 2000 to 2004, with each giving a percentage of 0.55% and 0.44%, respectively. Between 2000 and 2010, a total of 12 studies reported the prevalence of giardiasis in the range of 0.44%–24.9% (Nissapatorn et al., 2005; Al-Mekhlafi et al., 2005), while most of the 22 studies conducted between 2011 and 2020 among schoolchildren of the indigenous people reported a prevalence range of 0.5%–34.6% (Sahimin et al., 2020; Adli & Ghani, 2020).

**Table 1:** The reported cases of human giardiasis in Malaysia from 2000 to 2020.

**Num**	**Prevalence**	**Diagnostic method**	**Population**	**Reference**
1.	0.5% (*n* = 206)	Fecal microscopic and formalin ethyl acetate sedimentation	Urban poor	Sahimin et al. (2020)
2.	34.6% (*n* = 208)	Fecal microscopic and formalin-ether sedimentation	Indigenous schoolchildren	Adli & Ghani (2020)
3.	21.6% (*n* = 116)	Fecal microscopic of wet mount, trichrome staining and formalin ether sedimentation	Indigenous schoolchildren	Gee & Ghani (2020)
4.	12.1% (*n* = 473)	Fecal microscopic	Indigenous people	Noradilah et al. (2019)
5.	15.1% (*n* = 139)	Fecal microscopic and formalin-ether sedimentation	Indigenous schoolchildren	Jeyaprakasam & Abd Ghani (2019)
6.	10.8% (*n* = 388)	Fecal microscopic, formalin ether sedimentation, and nested PCR	Migrant workers	Sahimin et al. (2018)
7.	6.7 (*n* = 269)	Fecal microscopic and PCR	Indigenous people	Lee et al. (2017)
8.	11.5% (*n* = 340)	Fecal microscopic, trichrome staining	Children	Elyana et al. (2016)
9.	14.2% (*n* = 186)	Formalin-ether sedimentation technique and semi-nested PCR	Indigenous people	Chin et al. (2016)
10.	18% (*n* = 611)	Fecal microscopic, and nested-PCR	Indigenous people	Anuar et al. (2015)
11.	0.3% (*n* = 294)	Fecal microscopic, formalin-ether sedimentation and iodine staining	Inmates	Angal et al. (2015)
12.	28.3% (*n* = 498)	Fecal microscopic, formalin-ether sedimentation, and trichrome stain	Indigenous schoolchildren	Al-Delaimy et al. (2014)
13.	11.6% (*n* = 1330)	Wet mount and formalin-ether sedimentation	Indigenous people	Choy et al. (2014)
14.	18% (*n* = 611)	Fecal microscopic, formalin-ether sedimentation, trichrome staining, and nested-PCR	Indigenous people	Anuar et al. (2014)
15.	1.8% (*n* = 342)	Fecal microscopic and formalin-ether sedimentation	Children	Sinniah et al. (2014)
16.	17% (*n* = 484)	Fecal microscopic and semi-nested PCR	Indigenous schoolchildren	Huey et al. (2013)
17.	21.8% (*n* = 307)	Fecal microscopic and formalin ethyl acetate sedimentation	Indigenous schoolchildren	Al-Harazi, Abd Ghani & Othman (2013)
18.	22.2% (*n* = 374)	Fecal microscopic, formalin–ether sedimentation and trichrome staining	Indigenous schoolchildren	Al-Mekhlafi et al. (2013)
19.	20% (*n* = 500)	Fecal microscopic, formalin–ether sedimentation and trichrome staining	Indigenous people	Anuar et al. (2012)
20.	5.2% (*n* = 77)	Fecal microscopic and formalin-ether sedimentation	Indigenous people	Sinniah et al. (2012)
21.	10.4% (*n* = 716)	Fecal microscopic and formalin-ether sedimentation	Indigenous people	Ngui et al. (2011)
22.	5.7% (*n* = 122)	Fecal microscopic and nested-PCR	HIV/AIDS patients	Lim et al. (2011)
23.	3.2% (*n* = 346)	Fecal microscopic and formalin-ether sedimentation	HIV/AIDS patients	Asma et al. (2011)
24.	17.8% (*n* = 292)	Fecal microscopic, trichrome staining	Indigenous schoolchildren	Al-Mekhlafi et al. (2010)
25.	23.7% (*n* = 321)	Fecal microscopic, trichrome staining and nested PCR	Indigenous people	Mahdy et al. (2009)
26.	0.55% (*n* = 9992)	Fecal microscopic of wet mount and trichrome staining	^a^Inpatients	Mohamed et al. (2008)
27.	17.8% (*n* = 241)	Fecal microscopic, trichrome staining	Schoolchildren	Al-Mekhlafi et al. (2008)
28.	29.2% (*n* = 130)	Fecal microscopic, trichrome staining	Indigenous people	Yusuf et al. (2007)
29.	17.6% (*n* = 79)	Fecal microscopic, antigen test kit, trichrome staining and formalin-ether sedimentation	Inpatients and Indigenous people	Hakim et al. (2007)
30.	17.1% (*n* = 316)	Fecal microscopic, trichrome staining	Indigenous people	Mahdy et al. (2007)
31.	24.9% (*n* = 281)	Fecal microscopic, trichrome staining	Indigenous children	Al-Mekhlafi et al. (2005)
32.	0.44% (*n* = 1350)	Fecal microscopic and concentration technique	^a^Inpatients	Nissapatorn et al. (2005)
33.	6.92% (*n* = 159)	Fecal microscopic, trichrome staining	Indigenous people	Ghani, Kasim & Lai (2002)
34.	1.5% (*n* = 330)	Fecal microscopic and formalin-ether sedimentation	Indigenous people	Sagin et al. (2002)
35.	22.2% (*n* = 374)	Fecal microscopic, trichrome staining	Indigenous schoolchildren	Al-Mekhlafi et al. (2001)

**Notes.**

a Retrospective study based on medical records.

Studies in the population of Orang Asli focused on the west coast states of Peninsular Malaysia namely Selangor, Negeri Sembilan, and Perak, in addition to Terengganu and Pahang; the latter has the highest Orang Asli population in Malaysia, including the indigenous people of the east coast of Sabah and Sarawak. Surveys among the Orang Asli highlighted several factors contributing to *Giardia* infection, with main reasons being unsafe water resources, low hygienic practice, poor drainage system, socioeconomic level, large family members, seasonality, and geographical location (Yusuf et al., 2007; Ngui et al., 2011; Al-Delaimy et al., 2014; Chin et al., 2016). Researchers noted several common unhygienic habits such as eating raw fruits and sugarcanes without washing, preference to defecate near river streams instead of proper toilets, improper waste disposal, and lack of knowledge to utilize basic facilities provided by the government as part of the contributing factors (Ghani, Kasim & Lai, 2002; Anuar et al., 2012). Meanwhile, seasonal trend has also been linked to giardiasis where the case percentage is higher during wet season as compared to dry season. Heavy flood may have drained *Giardia* parasites from the environmental surfaces hence lower the risk of acquiring giardiasis during the dry season (Noradilah et al., 2019). Meanwhile, the contamination of rivers and lakes by *Giardia* could have been contributed by domestic and wild animals, while in areas with basic facilities of piped water supply, electricity, and toilets, this could have been due to contaminated water supplies, unhygienic lifestyle, and overpopulation.

Central to the study is the finding of a demographic fractions that shows children are an age group at higher risk of acquiring the infection, with a significant increment of prevalence observed in children less than 12 years old (Al-Harazi, Abd Ghani & Othman, 2013). Considering this, surveys on children aged 13 and below have been extensively carried out involving schoolchildren of Orang Asli and those living in urban cities. Of the study population, 62.9% of respondents were found positive for *Giardia* with over half of those surveyed coming from lower socioeconomic group, while only minorities came from increased family income (Rajeswari, Sinniah & Hussein, 1994). This is similar to the report by Shekhar, Prathapa & Gurpreet (1996) where only 0.21% (*n* = 7557) of primary schoolchildren with necessities of clean water supply and proper toilet were found positive, while the prevalence of giardiasis recorded among schoolchildren of the indigenous people was 24.9% (*n* = 281), with a slightly higher prevalence in children aged 2–7 years (Al-Mekhlafi et al., 2005). Further, about 23.42% (*n* = 111) of the indigenous children in Pahang were positive for *Giardia*, whereas only 14.7% (*n* = 456) of the schoolchildren in Gombak, a semi urban city in Selangor, were infected with giardiasis (Rajeswari, Sinniah & Hussein, 1994; Ghani & Musa, 2018). Undoubtedly, younger children are less susceptible to the infection due to their low immunity level, in addition to having low hygiene standards. Lifestyles or daily activities related to age and the presence of infected family members were the risk factors for giardiasis among children, where person-to-person contact within the family members was the possible mode of transmission. Several lines of evidence suggest that giardiasis is significantly linked with protein–energy malnutrition (PEM), micronutrient deficiency, particularly vitamin A deficiency (VAD), and iron deficiency anemia (IDA), all of which are predictors of malnutrition, stunting and wasting, which in turn lead to increased susceptibility to other infections, impaired cognitive function, and poor educational performance. This is supported by the findings from an anthropometrics measurement of Orang Asli children in Selangor where giardiasis was found as the predictor of stunting and wasting, suggestive that the nature of PEM among this population is of chronic and long duration (Al-Mekhlafi et al., 2005). In a population of 374 Orang Asli children in Pahang, 83 (22%) were infected with *Giardia*, and the prevalence of severe underweight, stunting and wasting was found to be 28.3%, 23.8%, and 21.0%, respectively (Al-Mekhlafi et al., 2013).

In a cross-sectional study among 241 indigenous schoolchildren in Pahang, 17.8% of the children were microscopically positive for giardiasis and 27.4% had low serum retinol levels below 0.70 µmol/L, an indicator of clinical VAD (Al-Mekhlafi et al., 2008; Al-Mekhlafi et al., 2010). Importantly, vitamin A is important for the normal functioning of the visual system and for maintaining epithelial cellular integrity. Therefore, vitamin A deficiency (VAD) can cause xerophthalmia, a condition of childhood blindness that affects 2.8 million children in developing countries (Feroze & Kaufman, 2021). It is also proven that giardiasis (and ascariasis) was significantly associated with low serum retinol concentrations and that the absorption of vitamin A is impaired in children infected with these infections.

On a different view, several researchers studied the distribution of genotype and subgenotypes of *Giardia* by performing multilocus genotyping (MLG) based on the genetic loci of SSU rRNA, triosephosphate isomerase (tpi), glutamate dehydrogenase (gdh), and β-giardin genes to observe the prevalence of difference assemblages, and to find the correlation between genetic assemblages with clinical symptoms. In an analysis of tpi gene by *Anuar et al. (2014)*, a prevalence rate of 18% was reported among 611 of Orang Asli from 8 villages in Negeri Sembilan, Perak and Pahang, with the presence of *G. duodenalis* assemblage A in 10.1% and assemblage B in 5.9% of the subjects (Anuar et al., 2014; Anuar et al., 2015). Interestingly in this study, assemblage A was reportedly significantly associated with gastroenteritis symptoms in subjects less than 15 years of age (77%). Meanwhile, another study noted the predominance of assemblage B among the Semai Orang Asli in Pahang, indicating the possibility of anthroponotic transmission of the protozoa in community (Mahdy et al., 2009). At the sub-assemblages level, most of the isolates of assemblage A found were from AII. This finding is similar to those reported by Huey et al. (2013), where AII was the predominant sub-assemblage (64%) found among 84 of the Orang Asli infected with giardiasis in Pahang (Huey et al., 2013). Although attempts have been made to determine the subtype of assemblage B, they have been unsuccessful due to the high nucleotide polymorphisms found in the isolates; hence, a more precise tool is needed to differentiate assemblage B at subgroup levels. The finding of assemblage B and the anthroponotic genotype AII implicates human-to-human transmission as the most possible mode of transmission among the indigenous communities in Malaysia.

Further, the prevalence of giardiasis was also studied in the urban and semi-urban cities of Malaysia, specifically in the state of Selangor. The increase in labor demand in multiple employment sectors arose in tandem with major urbanization experienced in Malaysia since the 1970s. Mass migration of workers from neighboring endemic countries inadvertently brought in various infectious diseases for which screening particularly for parasitic diseases is inadequate (Sahimin et al., 2016). Migrant workers are among the 3.8% of the population living below the poverty line, occupying overcrowded housing areas that lack sanitation and clean water facilities, promoting the widespread of the disease (Abdul-Aziz, 2001). A study by Sahimin et al. (2018) reported a prevalence rate of 10.8% (30/388) among these workers and identified assemblage B to be predominantly present in 56.7% (17/30) of the population, whereas 13 of them were classified as *G. duodenalis* assemblage AII (13/30; 43.3%) (Sahimin et al., 2018). The fact that most of cases were reported among the newly arrived workers, suggests that the infections had been acquired most likely in their country of origin, rather than after their arrival in Malaysia.

Other than studies in immunocompetent individuals, the risk of IPIs, including *Giardia* was also evaluated in population with immunosuppression, *i.e.*, inpatients and HIV-infected patients. In 1981, the prevalence of giardiasis cases in patients admitted to hospitals was 2.8%, which decreased to 0.7% in 2001 (Norhayati et al., 2003). The overall prevalence of IPIs among HIV patients was found to be considerably higher (37.9%, *n* = 346 patients), with 3.2% of them having giardiasis (Asma et al., 2011). A tpi–gene analysis revealed that four out of seven positive isolates from HIV patients were infected with *G. duodenalis* assemblage A. The rate is a bit higher as 5.7% of 122 HIV patients from two major teaching hospitals in Kuala Lumpur also had giardiasis, in which *G. duodenalis* assemblage A was identified in 4 samples (Lim et al., 2011). In a study investigating IPIs among prison inmates, only 0.3% (*n* = 294) had giardiasis from a total of 26.5% of IPIs (Angal et al., 2015). The low prevalence of giardiasis was also reported in a retrospective study of stool samples from patients attending HUSM from 2000 to 2006. Of the 9992 stool samples screened, only 55 cases of positive giardiasis were found, giving an overall prevalence of 0.55% (Mohamed et al., 2008), and 6 cases were retrospectively found in another study that reviewed 1,350 stool samples received from 2000 to 2004 (Nissapatorn et al., 2005. Table 1 summarizes the overall cases of giardiasis in humans that reportedly occurred in the country from 2000 to 2020.

### Prevalence of giardiasis in animals

Likewise, surveillance studies also discovered the simultaneous presence of *Giardia* in human–animal–environment. In the indigenous communities of Selangor, Pahang and Perak, *Giardia* cysts were found in 6.7% of the indigenous people, 4.7% among the pet animals, and at the average concentration of 0.1–5.97 cysts/L in river water samples (Lee et al., 2017). Of note, data from several animal studies suggest that the concentrations of *Giardia* cysts may reach >504 cysts/g in sheep, >6061 cysts/g in dogs, >7143 cysts/g in domestic cats and >16,667 cysts/g in pigs (Cox et al., 2005; Lee et al., 2017). From the surveillance studies in cattle farms located near Sungai Langat Basin, *Giardia* cysts were found in 14.6% (*n* = 96) of bovine fecal samples with a range of 75–1.3 ×10^4^ cysts/g, and 6.7% (*n* = 45) in cattle wastewater at a concentration of 4–75 cysts/ml (Farizawati et al., 2005).

The surrounding area had cysts detected in river water at densities of 1.3–9 cysts/L, a 22-fold times higher than the concentration of 0.4/L that was known to have caused outbreaks in Ayrshire, Swindon, and Bradford in the United Kingdom and Milwaukee in the United States (Karanis, Kourenti & Smith, 2007). Besides, 237 dogs in a housing estate area of Perak were tested for the presence of the infection, which in turn yielded a percentage of 21.9% of positive infection (Rahman, 1990). Meanwhile, 28.6% of domestic and wild rats caught in the community of Temuan Orang Asli were positive for *Giardia* cysts, at a range of 10–59 640 cysts/g, while 14.6% (*n* = 89) of wild rats captured in Serdang and 3% (*n* = 134) of urban rodents harbored *Giardia* cysts (Lim & Ahmad, 2004; Tan et al., 2019). A recent study by Tijjani et al. (2020) found *Giardia spp.* in 14.6% of 89 wild rats captured in the area of Universiti Putra Malaysia. *Lim et al. (2009)* reported the presence of Giardia cysts in water bodies from a Malaysian zoo, ranging from 1 to 120 cysts/l identified at all sampling sites. Further, a total of 310 goats from 8 different farms in 4 states of Malaysia were screened for *Giardia spp.* and found the “zoonotic” assemblages A in 3 samples and assemblage B in 1 sample, suggesting that there might be cross-transmission from ruminants to humans (Lim et al., 2013). Although assemblage E is the predominant genotype in goat and might be less risky to humans, the presence of assemblages A and B in this animal implies that the goat infected with assemblage A/B posed serious threat to humans and is of clinical importance as they can be zoonotic reservoirs of the infection.

### Prevalence of giardiasis in environment

Meanwhile, aquatic biomonitoring of *Giardia* cysts in river water in five rural villages in Selangor, Pahang and Perak showed the presence of cysts in 51.3% of samples (20/39), with an overall mean concentration of 0.10–25.80 cysts per liter of water (Lee et al., 2014). It has been observed that river contamination with cysts in Orang Asli areas could be possibly due to defecation near rivers or cysts from contaminated land areas being washed away in heavy rain and flood into the river, resulting in heavy environmental contamination.

What is more important is the finding of *Giardia* in 90% of the raw water samples from 2 drinking water plants in Selangor, and 51.6% of raw water samples from 3 water treatment plants in Negeri Sembilan, each at the levels of 100–2,140 cysts/100 L, and 0–12 cysts/L, respectively (Ahmad et al., 1997). While no *Giardia* cysts were detected in the treated water samples from these sites, *Giardia* cysts were found in one treated sample and in 32.9% of raw water samples isolated from two drinking water treatment and seven distribution system sites in Sarawak (Richard et al., 2016). However, it is perceived that the presence of *Giardia* cysts in the treated water may not be due to treatment failure but rather due to minimal treatment process which was insufficient to eliminate protozoa. Additionally, *Giardia* was also found in 17.9% of well water samples and at about 0.4–2 cysts per liter in household water stored in buckets, a source of water that is most likely to be used as potable drinking water (Lim & Ahmad, 2004; Ahmad & Chan, 1994). The presence of *Giardia* cysts was also reported in recreational waters collected from Sungai Congkak (50%, *n* = 30) and Sungai Batu (16.6%, *n* = 30), both located in Selangor (Ithoi, 2009). Above all, *Giardia* cysts have been observed at 16.4–66.7% of occurrence rates in river systems in Malaysia (Ahmad & Chan, 1994).

A significant analysis and discussion on waterborne parasite occurrences, focusing on *Giardia* and *Cryptosporidium* cases in various types of water samples in ASEAN countries, including Malaysia was thoroughly reviewed by Lim & Nissapatorn (2017), Lim & Nissapatorn (2017). Drawing from various reference sources, it can be concluded that the presence of *Giardia* in all types of water samples, most often in untreated and rarely in treated water, is closely related to the presence of human settlements, agriculture/livestock, and human activities such as defecation or waste disposal near rivers, demonstrating the importance of more epidemiological studies to be conducted to better understand the transmission of giardiasis in the human–animals–environment interface in compliance with the One Health concept.

## Conclusions

Thus far, the outbreak of giardiasis in Malaysia has never been reported and is also not listed in the notifiable diseases gazette by the Ministry of Health (MOH), suggesting that the true prevalence of giardiasis in the country tended to be underreported. The lack of standardized reporting systems, in addition to inadequate routine monitoring and coordinated efforts to collect epidemiological data of *Giardia* infection, and less sensitive detection methods add to the paucity of surveillance data. The important fact is that giardiasis is more prevalent among the poorest segments of the population, the indigenous people and densely populated areas such as slums and prisons, where unhygienic lifestyle and behaviors, limited sanitation, and safe water facilities in this population add to the challenge of reducing giardiasis cases in this country.

The Sustainable Development Goal 3 (SDG 3) aspires to ensure healthy lives and promote well-being for all at all ages, in which one of the specific highlights is to reduce mortality of the under-5 to as low as 25 per 1,000 live births and to end the epidemics of waterborne disease. Despite great advances in the socioeconomic status of Malaysia, giardiasis remains a public health problem mainly among Orang Asli children in poor rural areas. Although the last 10 years has shown that the overall mean rate of giardiasis in Malaysia is quite encouraging and appears to be declining at 13.7%, there has been a slight increase in the prevalence of underweight, stunting, and wasting particularly among rural children.

According to the statistic of environmental health for rural area in 2018, the rate of houses in rural areas served with safe water supply and sanitary latrines accounts to 95.89% and 95.92%, respectively, yet there is still an increase in the prevalence of underweight, stunting, and wasting of rural children from 14.9%, 16.8%, and 7.2% in the year 2012 to 15.6%, 22.2%, and 8.5% in 2019, respectively (National Institutes of Health Ministry of Health Malaysia, 2020a; National Institutes of Health Ministry of Health Malaysia, 2020b). In line with this, the Malaysian Global Nutrition Targets 2025, outlined as part of the National Plan of Action for Nutrition of Malaysia III (2016–2025), has also specified a target to achieve 40% reduction in the number of children under 5 years who are stunted. As giardiasis is known to be associated with long-term developmental problems of stunted growth and malnutrition, as well as one of the leading causes of diarrhea-related mortality in children, addressing and providing better disease control against giardiasis should be a priority, and this is well aligned with the current government and international policy to provide better quality of life to children.

Giardiasis infection can be prevented with clear cognizance and can be treated well if it is accurately diagnosed. Integrated efforts from various agencies such as clinicians, health providers and researchers coupled with political commitment and public awareness are very much important in reducing transmissions and mitigating the infection. Access to improved drinking water sources through piped connection or other sources including public taps, protected wells, and boreholes should be extended to all areas, while community-based education should be disseminated to all levels to increase public awareness and understanding on the importance of treatment and hygienic lifestyle to curb giardiasis. Surveillance system should be strengthened with case notification and the frequency of epidemiological studies coupled with periodic preventive chemotherapy should be increased to assist in the formulation of prevention and control measures. In line with this, support for research should be enhanced to improve the diagnostic accuracy of giardiasis. All these are the crucial part of efforts in the control and prevention of giardiasis.

## Additional Information and Declarations

### Data Availability

The following information was supplied regarding data availability: The raw data is available in Table 1.
